# Antibiofilm Properties of Silver and Gold Incorporated PU, PCLm, PC and PMMA Nanocomposites under Two Shear Conditions

**DOI:** 10.1371/journal.pone.0063311

**Published:** 2013-05-13

**Authors:** Shilpa N. Sawant, Veerapandian Selvaraj, Veluchamy Prabhawathi, Mukesh Doble

**Affiliations:** 1 Chemistry Division, Bhabha Atomic Research Centre, Mumbai, India; 2 Department of Biotechnology, Indian Institute of Technology, Chennai, India; Consejo Superior de Investigaciones Cientificas, Spain

## Abstract

Silver and gold nanoparticles (of average size ∼20–27 nm) were incorporated in PU (Polyurethane), PCLm (Polycaprolactam), PC (polycarbonate) and PMMA (Polymethylmethaacrylate) by swelling and casting methods under ambient conditions. In the latter method the nanoparticle would be present not only on the surface, but also inside the polymer. These nanoparticles were prepared initially by using a cosolvent, THF. PU and PCLm were dissolved and swollen with THF. PC and PMMA were dissolved in CHCl_3_ and here the cosolvent, THF, acted as an intermediate between water and CHCl_3_. FTIR indicated that the interaction between the polymer and the nanoparticle was through the functional group in the polymer. The formation of *E.coli* biofilm on these nanocomposites under low (in a Drip flow biofilm reactor) and high shear (in a Shaker) conditions indicated that the biofilm growth was higher (twice) in the former than in the latter (ratio of shear force = 15). A positive correlation between the contact angle (of the virgin surface) and the number of colonies, carbohydrate and protein attached on it were observed. Ag nanocomposites exhibited better antibiofilm properties than Au. Bacterial attachment was highest on PC and least on PU nanocomposite. Casting method appeared to be better than swelling method in reducing the attachment (by a factor of 2). Composites reduced growth of organisms by six orders of magnitude, and protein and carbohydrate by 2–5 times. This study indicates that these nanocomposites may be suitable for implant applications.

## Introduction

Metallic, ceramic and, metal nanoparticles are added to polymers to obtain unique physical and mechanical properties which cannot be achieved by adding micron-sized particles. The extent of modification of the property depends on the base polymer, the size, distribution and dispersion of the nanoparticles and on the adhesion at the filler-matrix interface [1]. A nanoparticle dispersed in polymer is called polymer nano composite and it is considered as a single homogeneous material. These materials exhibit unique thermal, mechanical, and biological properties when compared to conventional composites [2], [3].

Silver and gold nanoparticles show antibacterial activity [4]. Generally they are prepared by chemical and biological methods. Chemical preparations are widely studied because of their ease and wide applications. Several nanocomposites have been reported by using various nanoparticles and base polymers including PCL, PU, and PP [5], [6], [7]. Silver, gold and copper nanoparticles are reported to exhibit strong biocidal effect on more than sixteen species of bacteria including *Escherichia coli* [8], [9], [10], [11], [12]. Nanoparticles have an extremely large relative surface area to volume, and hence increasing their contact with bacteria or fungi, vastly improve their bactericidal and fungicidal effectiveness. They bind to microbial DNA, preventing bacterial replication, and to sulfhydryl groups in the metabolic enzymes of the bacterial electron transport chain, causing their inactivation [12], [13].

The studies of microbial biofilms are necessary in different areas including medicine [14], [15], food processing industries etc [16]. *E. coli* is a pathogenic bacteria reported in food and water contamination and medical implants [17], [18] and aquatic environment [19]. They colonize the gastrointestinal tract of human and cause a broad spectrum of diseases.

*E. coli* is the predominant organism involved in Urinary tract infections and it also leads to antibiotic resistance [18]. It forms biofilm in implants placed in the urinary region causing infection, inflammation and hence its rejection.

Biofilm is difficult to eradicate since it provides protection for the micro organism from the host immune system and antimicrobial therapies [14]. Antibiotic therapy is often administered during the implantation stage as high doses. This practice, can lead to adverse drug reactions as well as produce resistant microorganisms. Better infection prevention strategies include the use of antibiotic or antimicrobial coatings on device surfaces [20], [21] and impregnation of device components with antibacterial silver [22].

Coatings are usually effective for short-term applications. There is always need for research to design better antibacterial biomaterials for long-term use. This study investigates the preparation and testing of nanocomposite (NC) to achieve long term antibacterial functionality. Use of passive coatings that alter the physiochemical properties of the substrate and coatings that actively release antibacterial agents are widely used. While the former including hydrophilic polyurethanes [23] reduce bacterial adhesion, they provide no alternate to kill bacteria which adhere to the polymer. Low levels of adhered viable bacteria may of eventually build up and lead to implant infection. So it is desirable to develop coatings that are also capable of killing adhered bacteria.

Here we report for the first time the preparation of silver and gold nanoparticle in the presence of a cosolvent. Different types of polymers are used as biomaterial for various applications [24]. Present paper is a broad investigation which studies the effect of gold and silver nanoparticle as an antibacterial agent on various polymers. Four nanocomposites are prepared with these two nanoparticles as fillers using polyurethane (PU), polycaprolactam (PCLm), polycarbonate (PC) and Polymethylmethaacrylate (PMMA). These polymers are chosen for the current studies since they have different functional groups present and are expected to exhibit different interactions with these two metallic nanoparticles. These polymers are also widely used in medical implants, food storage vessels etc. Polyurethane is known for its biocompatibility and rubber like properties and hence used for various biomedical applications [14]. Acrylic polymers are widely used, as bone cement, in the treatment of bone defects and prosthetic and as orthodontic material [15]. They degrade due to the formation of biofilms. PC, due to its mechanical strength is used in bone replacement. PCLm is used in blood contacting area [21]. Polycarbonates are hydrophobic with a low free energy surface. So making the surface more hydrophilic can reduce the formation of biofilm. There are numerous studies on nanocomposites which are prepared by swelling [25], [26] and casting methods [27], [28]. But there are no studies which compare the effectiveness of both these methodologies in terms of their antibacterial activity. Present study focuses on this aspect.

Nanocomposite (NC) of polyurethane (PU) using glass and silicate has been widely reported [23], [29]. *S. epidermidis* adhesion on PU nanocomposite has been reported [30]. Performance of chitosan/organic rectorite NCs [31] and polydimethyloxane (PDMS)/clay–silver–chitosan NC have been studies with a range of urinary pathogens [32].

Biofilm experiments are generally done using shake flasks at fixed rpms. But in real environment, biofilm forms both in high and low shear conditions. Biofilm grown in high shear conditions include streams, waterlines or a shore line exposed to wave action [33] or cardiovascular or ureteral region. Low shear environment includes catheters, food processing conveyor belts, lungs, cystic fibrosis and the oral cavity [34]. Depending upon the shear conditions, biofilm growth differs. So, it is necessary to understand this process at different shear conditions. So, in the present study, the antibiofilm effect of different nanocomposite is studied against *E. coli* by using Drip flow Biofilm Reactor (DFBR) and Shaker (180 RPM). The former produces low shear and the latter high shear near the polymer surface. Shear forces are known to affect the morphology and growth of bacteria and the extent of attachment to surfaces [35]. Implants experience fluids flowing at different flow rates and surface shears depending upon its location in the human body. For example, the blood flow is almost zero near bends and constrictions which is ideal for proteins and microorganisms to settle, whereas the flow rates are high near the heart. Hence the performances of these nanocomposites were also studied at two different shear forces in order to determine whether these modifications perform well at both these conditions.

## Experimental Section

### Materials

All the polymers were supplied by Industrial and marine suppliers Company, Chennai, India and the chemicals by Merck, India. *Escherichia coli* (*E. coli*) NCIM 293 was purchased from National Chemical Laboratory (NCL), Pune, India. It was stored in glycerol stock at −20°C and used when required.

### Preparation of Nanoparticles

A 50% solution of AgNO_3_ (50 ml of 3 mM) in THF was refluxed for 5–10 mins and an aqueous solution of NaBH_4_ (50 ml of 3 mM) was added to it. Reflux was continued until a yellowish [36] solution was obtained (approximately after 30 mins). This solution was passed through a 0.45 µm Millipore syringe filter to remove any precipitate, and the filtrate was stored at room temperature.

A 50% solution of HAuCl_4_ (19 ml of 4 mM) in THF was refluxed for 5–10 min, and a warm (50–60°C) aqueous solution of sodium citrate (1 ml of 0.5%) was added to it quickly. Reflux was continued for another 30 min until a deep-red [37] solution was observed. The slurry was passed through a 0.45 µm Millipore syringe filter to remove any precipitate, and the filtrate was stored at room temperature.

### Polymer Modification

Nanocomposite were prepared by two methods as described below.

#### Modification of polyurethane and polycaprolactum

PU and PCLm are soluble in THF, so the incorporation of the nanoparticles adopted for these two polymers are the same.

In the swelling method [38], [39] the silver nanoparticles solution (10 ml) is poured on the polymer film (5 cm^2^) which is placed in a Teflon plate. Since the solution has THF, the polymer gets swollen and the nanoparticles penetrate into it. After 24 hrs, the film is dried at 50°C and washed with Millipore water to remove weakly adsorbed nanoparticles from its surface. The same procedure is followed for incorporating gold nanoparticles as well.

In the casting method the Ag nanoparticles solution (10 ml) is mixed with the polymer solution (1 g of polymer dissolved in 10 ml THF) under stirring. The nanoparticle impregnated polymer is obtained as a gel, which separates as a composite. The mixture is then dried at 50°C and the gel is dissolved (20 ml) in THF. The solution is recast in a Teflon plate and dried at room temperature. The same procedure is followed for gold nanoparticles as well.

#### Modification of Polycarbonate and poly (methylmethaacrylate)

PC and PMMA are soluble in CHCl_3,_ so the modification procedure is the same for these two polymers. Here a 1∶1 mixture of THF and CHCl_3_ is used to prepare the nanocomposites by swelling and casting methods as described above.

### Characterizations of the Materials

The nanoparticles produced are characterized by (Jasco V 550) UV-Visible Spectroscopy and their morphology and size are measured by transmission electron microscope (JEOL 3010 UHR). The crystalline nature of the dry nanoparticles is confirmed by XRD (X ray diffraction) analysis (Philips PW 1830X-ray). The functional group in the polymer and the nanocomposites are determined by (Perkin-Elmer3100) Fourier Transform Infra Red (FTIR) Spectroscope by using KBr discs.

Scanning electron microscopic images of the Au nanocomposite is captured with a g scanning electron microscope (SEM), (Jeol JSM 5600 LSV model).

The nanoscale morphology of these films are measured with Veeco NanoScope IV Multi Mode AFM (Atomic Force Microscope) equipped with a heating accessory [40]. All measurements are done in tapping mode. The surface hydrophobicity of the polymers are determined by measuring the advancing and receding contact angles using a Kruss Easy drop goniometer (KRUSS, DSA II GmbH, Germany). Ultrapure water is used as the contact angle liquid.

### Bacterial Studies

The *E. coli* biofilm is grown on the four nanocomposites prepared by both the methods using low and high shear bioreactors. *E. coli* is grown in nutrient broth. The culture is incubated for 18 hrs in a shaker at 37°C until its optical density (OD) reached 0.65.

### Drip Flow Biofilm Reactor (DFBR)

The polymer strips (of 1×1 cm and 0.4 cm thick) are sterilized by dipping them in 70% ethanol for 24 hrs. A low shear biofilm is grown on the polymer in a drip flow reactor (Model DF 202, BioSurface Technologies Corp., Bozeman, MT) [41]. The reactor consists of four channels in which different sample strips are placed and the broth is made to flow in each channel by using a four chamber peristaltic pumps. The reactor is modified to accommodate rubber sheeting machined to hold one coupon (1 cm^2^) in each of the four channels. The polymer NC is pasted on to a borosilicate glass coupon. They are cleaned according to ASTM E2196-02 standard [29]. Experiment on biofilm formation was performed according to a reported methodology [42]. Each channel is initially inoculated with 1 ml of *E. coli* and 20 ml of broth and grown at 23±1°C for 6 hrs under static condition. Then, a constant flow of the medium (1 ml/min) containing the organism (*E. c*oli) is passed over the polymer film for next 6 hrs. After the experiment the polymer film is removed from the glass using sterile forceps and washed twice with 0.85% saline to remove loosely adhered bacteria. The strongly bound microbes are then removed from the polymer surface by waterbath ultrasonication (THOSAN Pvt. Ltd., Ajmer, India) (total of 10 min with 1 min intervals) and the number of viable colonies are counted visually in tryptic soy agar plates [43].

### High Shear

A high shear biofilm is grown in a conical flask agitated in an orbital shaker (Shigenics, India). Each conical flask contains several polymer pieces of 1 cm^2^ area. 20 ml of the nutrient broth is inoculated with 1 ml of *E. coli* solution (grown as described above) into the flask and the experiments are conducted at 180 RPM for 6 hrs.

The total carbohydrate (Phenol sulfuric acid method) [22], protein (Bradford method) [43] and Colony forming unit (CFU or live cell attached) on the polymer surface [44] are measured on three separate samples and the average of the results are presented here.

### Statistical Analysis

SPSS ver 15.0 was used to perform ANOVA and two sample t-test. A p value <0.05 is considered as statistically significant. All the bacterial studies were repeated thrice and the average values with standard deviations are reported here.

## Results and Discussion

### Characterisation of Nanoparticles

**Figure 1:**
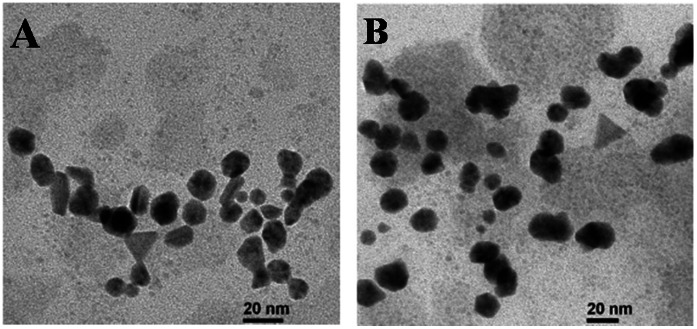
TEM images of (A) Silver and (B) Gold nanoparticles.

Strong surface plasmon resonance for Ag nanoparticle is centered at 418 nm (Figure S1 in File S1) and such a peak is reported for various metal nanoparticles, with sizes ranging widely from 2 to 100 nm [32], [45]. Similarly a strong surface plasmon resonance for Au nanoparticle centered at 550 nm (Figure S2 in in File S1) is observed [43]. Figure 1 shows representative TEM images of both the nanoparticles. The images show that nearly spherical shaped particles are more abundant, with are average size of ∼20–27 nm.

**Figure 2:**
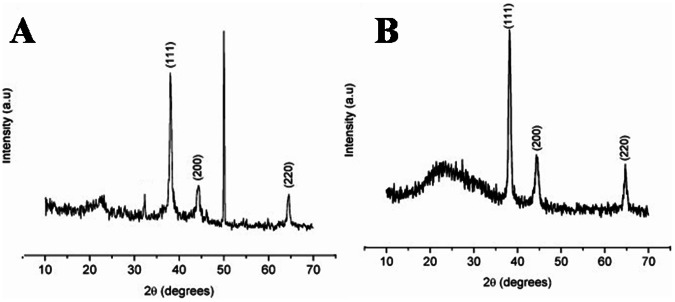
X-Ray Diffraction of (A) Silver and (B) Gold Nanoparticles.

Figure 2 shows the XRD pattern of Ag and Au nanoparticles. The diffraction peaks at 38.1°, 44.5° and 64.6° correspond to (1 1 1), (2 0 0) and (2 2 0) planes respectively [46], [47] of the face centered cubic crystal structure. In figure 2A, the peak at 50° corresponds to impurity. The peak corresponding to the (1 1 1) plane is more intense than the other planes. The ratio between the intensity of the (2 0 0) and (1 1 1) diffraction peaks is much lower than the usually reported value (0.52) suggesting that the latter plane is the predominant orientation [48]. The width of the (1 1 1) peak is employed to calculate the average crystallite size using Scherrer equation [49]. The calculated average size is ∼ 27 nm which matches with the particle size obtained from TEM images for both the nanoparticles.

### Characterization of Nanocomposites

**Figure 3:**
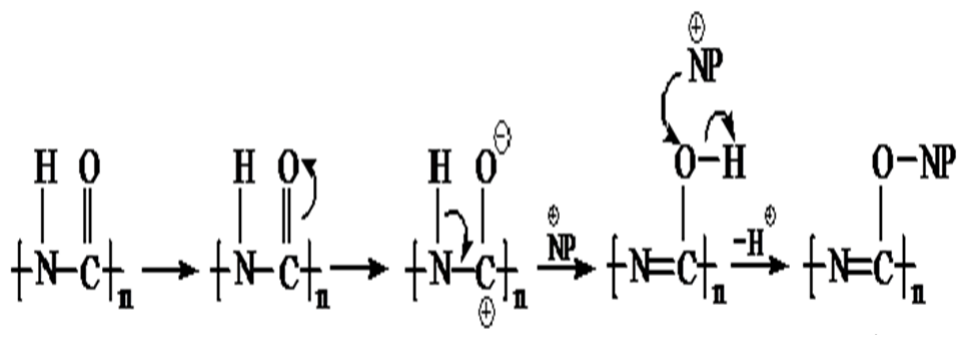
Movement of charge during the incorporation of nanoparticle in PU and PCLm. **NP = Nanoparticle.**

**Figure 4:**
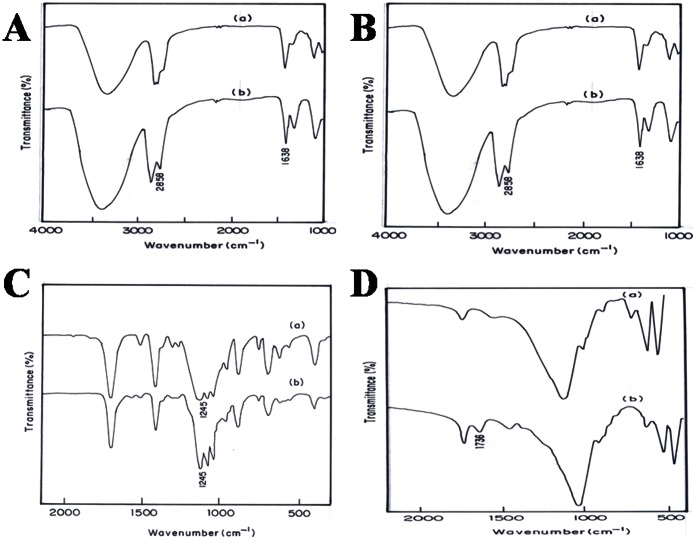
FTIR spectra of virgin polymers (a), and their corresponding silver nanocomposites (b), prepared by casting method. **(A) PU; (B) PCLm; (C) PC; and (D) PMMA.**

The FTIR spectrum of the PU (silver casting) nanocomposite reveals a few new peaks which are not present in the virgin polymer. Modified polyurethane has a peak at 1638 cm^−1^, representing C = N frequency [50], which is not seen in the virgin polymer. The functional group in PU is, NH-CO-, and the virgin polymer does not have C = N. During the modification, the carbonyl group is probably polarized (see Figure 3), followed by the movement of H to O atom. So N forms a double bond with C atom. Hence the modified polyurethane shows a peak corresponding to C = N. The nanoparticles later replace the H atom. The peak at 2858 cm^−1^ indicates the CH_2_ stretching frequency. C = N affects the nearest –CH_2_ group, so a peak appears at 2858 cm^−1^. The FTIR of polycaprolactam (Figure 4B) is similar to that of polyurethane. So the nanoparticle in this case probably also binds to the polymer in the same way (Figure 3).

**Figure 5:**
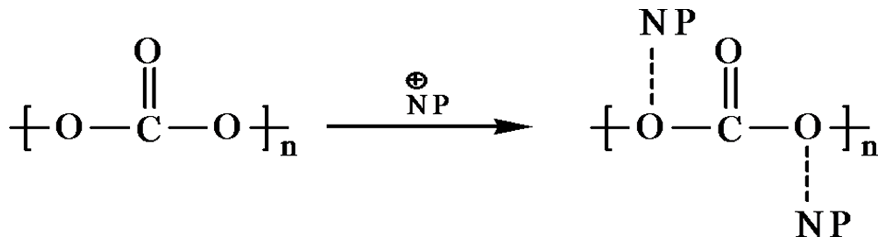
Chemical interaction of nanoparticle with polycarbonate.

The carbonyl group in PC is not polarized, since the –C = O group has –O- atoms on either side. The electronegativity of O atoms pulls the electron from the C atom on all the three sides. The peak corresponding to –C-O-C- at 1245 cm^−1^(Figure 4C) in the modified polymer is split [51]. This peak split indicates that the nanoparticle is attached weakly with –O- atom as indicated in figure 5.

**Figure 6:**
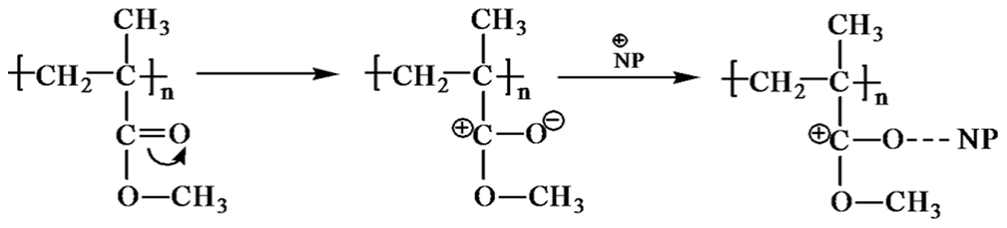
Bonding of nanoparticle to PMMA.

Weak bonding of NP to the O in the carbonyl group (Figure 6) in PMMA is confirmed by FTIR spectroscopy. Figure 4D shows the appearance of two characteristic peaks at 1736 and 2955 cm^−1^ which are assigned to carbonyl group and stretching vibration of C–H in PMMA structure, respectively [50].

So, the FTIR spectra indicate that the Ag nanoparticle is bound to each of the polymer via the functional groups present in the latter. The FTIR spectra of the four gold-polymer nanocomposites are present in the supporting information (figures S3 - A to D in in File S1). We see a similar interaction between the gold nanoparticle and the polymer.

**Figure 7:**
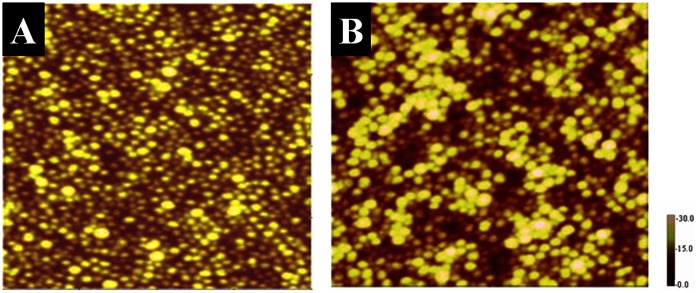
AFM tapping mode images of silver PC nanocomposite prepared by (A) casting method and (B) swelling method,(scan size 1 µm × 1 µm, surface roughness scale in nm).

**Figure 8:**
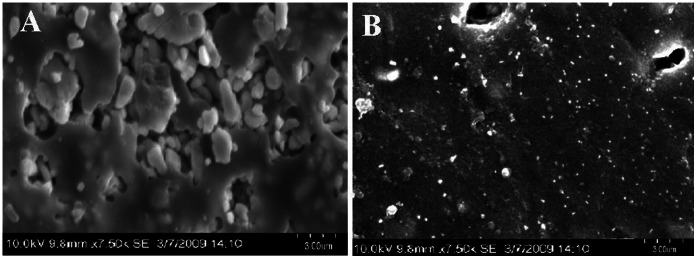
SEM images of PC surface (A) unmodified and (B) Au modified prepared by casting method.

Figure 7 shows the AFM images of PC nanocomposite with silver nanoparticle by the casting and swelling methods respectively. The nanoparticles are well separated in the former method, where as the particles appear agglomerated in the latter method, thereby loosing its efficiency. After the modification, the surface roughness of the polymer is reduced from 30 to 15 nm and highly planer. Figure 8 shows the SEM images of unmodified and gold modified (by casting method) PU surfaces. It is clearly seen that nanocomposite surface is more uniform and smooth when compared to unmodified surfaces. These images indicate that addition of nanoparticle improves the surface topography. PU is the most hydrophilic (77.2°), followed by PCLm (83.0°) and then PMMA (84.3°). PC is the most hydrophobic (88.9°) in this set of four polymers.

### Antibiofilm Property of NCs

**Figure 9:**
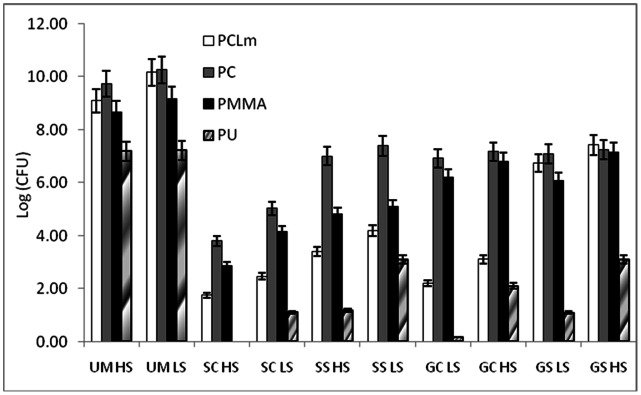
Effect of various treatment strategies on the amount of live colonies present in the biofilm on the surface. PU = Polyurethane, PC = Polycarbonate, PCLm = Polycaprolactum and PMMA = Poly(methylmethaacrylate), HS = High Shear, LS = Low Shear, GC = Gold Casting, GS = Gold Swelling, SC = Silver Casting, SS = Silver Swelling.

**Figure 10:**
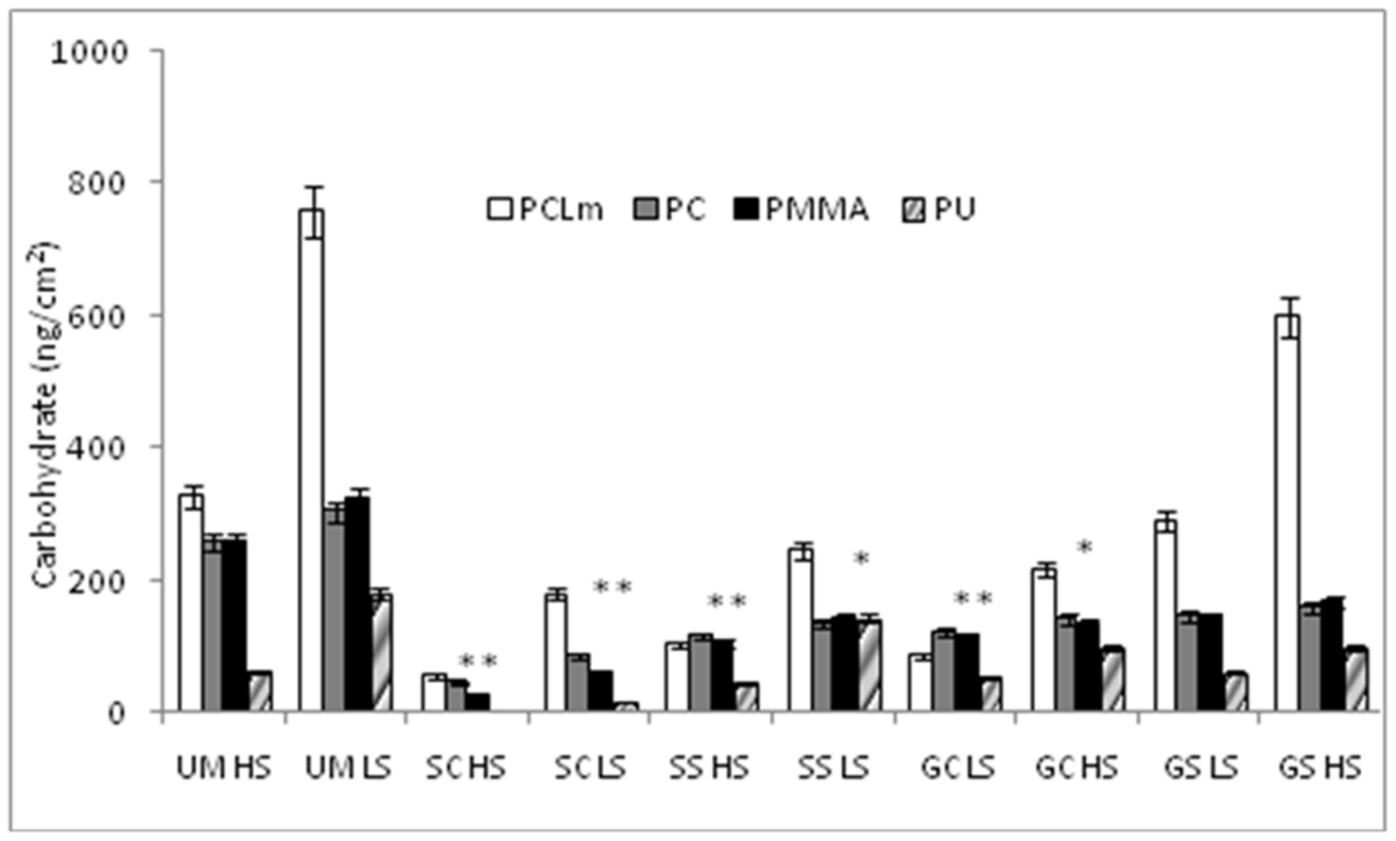
Effect of various treatment strategies on carbohydrate attached on the surface. **Legends same as in **figure 8****.****

**Figure 11:**
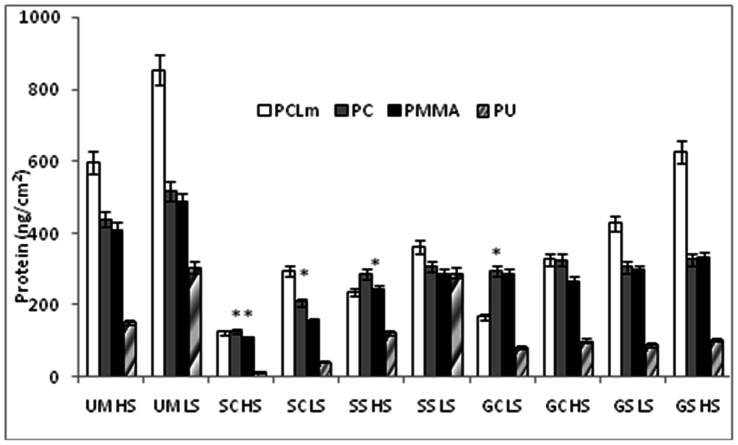
Effect of various treatment strategies on protein attached on the surface. **Legends same as in **figure 8****.****

The amount of live cells (CFU), carbohydrate and protein on various unmodified and modified polymers under high and low shear conditions are shown in Figures 9, 10 and 11 respectively. The shear forces encountered by *E. coli* under the present experimental conditions in the drip flow biofilm reactor and orbital shaker are approximately 0.15 and 2.5 Pa respectively. The carbohydrate and protein is measured from live as well as dead cells in the biofilm whereas the CFU indicates only live cells on the surface present at that point of sampling. All the silver modified surfaces reduce the amount of attached live cells by an order of four to seven. Gold modified surfaces reduce the amount of live bacteria by an order of two to six. The *E. coli* growth is low on silver modified surface when compared to gold modified surface since silver has high antibacterial activity than gold nanoparticles [43]. The amounts of protein and carbohydrates got reduced by 5–12 times on nanocomposites, indicating the superiority of these over the corresponding virgin counterparts. We have observed similar behavior with poly aniline and silver coated PU [22], ZnO and chalcone coated cotton fabric [52] and chalcone coated polymers [53] against wide range of clinical strains and marine bacteria. In all the cases here the carbohydrate, protein and CFU are high in the low shear conditions than in the high shear conditions. The shaking reduces the attachment, so high shear condition leads to low attachment. Bacterial adhesion to implanted medical devices depends also on the flow of body fluids. Attachment of *S. epidermidis* is low on high shear conditions [35]. Physical forces and shear generated by local flow dynamics may modulate the adhesion process. Experimental data required on the critical shear rate to prevent adhesion and to simulate detachment of already adhering microorganisms from glass and hydrophobic surfaces is reported by several researchers [54]–[56]. *E. coli* growth is low on polymer surface modified by casting than swelling method, probably because the nanoparticle is present more uniformly through out the polymer in the former. In addition agglomerate of the nanoparticles on the surface in the latter method (as seen in AFM) may also be reducing the efficiency of the composite.

A strong positive correlation exists between contact angle of the virgin polymer and attachment (CFU/ml) on the surface (corr coeff = 0.65–0.87). Attachment is lowest on PU. Attachment is more on hydrophobic surface (higher contact angle) than on hydrophilic surfaces. Such behavior has been observed in many previous studies [20], [56]. Contact angle is inversely related to surface energy. A high surface energy material is hydrophilic and will have low contact angle. Adhesion of *S. epidermidis* to He and He/O_2_ treated PET surface in a flow reactor is found to be negatively correlated to surface energy, matching well with our findings [35]. Gallardo-Moreno et al observed that ultraviolet irradiation of titanium alloy increased its surface free energy and reduced *S. epidermidis* adhesion. Initial conditioning film that is formed on the surface alters the subsequent attachment pattern and this will no longer depend on the contact angle of the virgin surface. In addition to surface hydrophobicity other parameters including flexibility, surface roughness, charge, additives etc may also play a role in determining the formation and various constituents of the biofilm on a polymer. We observed that nanocomposite reduced the surface roughness. PU is flexible and swells while PC is rigid and hence the former may prevent long term adhesion of biofilm. A positive correlation exists between contact angle and protein attached in most of the cases (correlation coefficient varies from 0.6 to 0.90). Similarly a positive correlation exists between contact angle and carbohydrate (0.7 to 0.92).

## Conclusion

In this study Ag and Au nanocomposite are prepared with four polymers commonly used in medical implants. The four polymers are dissolved either in THF or CHCl_3_. The nanocomposite are prepared by using water as a solvent and THF as cosolvent. The nanoparticles prepared are of uniform size (size ∼20–27 nm). The interaction between the polymer and the water is very low, while the cosolvent increases the interaction between the polymer and the nanoparticle. Both the modification methods reported here are very simple. SEM and AFM images indicate that after the modification (with Au and Ag) the polymer morphology has changed, and its surface has become smoother and closely packed. FTIR indicates that the interaction between the polymer and nanoparticles (Au or Ag) are mainly with the functional group present in the former. The effects of carbohydrate, protein and the Bacteria interaction with unmodified and modified polymers have been studied. The *E. coli* growth is reduced by 10^6^ times on modified surfaces and reduction is higher on the silver modified surface, since it is a known antibacterial when compared to gold modified one. More biofilm growth is seen in drip flow biofilm reactor when compared to growth in shaker (the former offers approximately ten times less shear than the latter). This study also indicates that biofilm growth on a surface may double if the shear reduces by a factor of 15.

The hydrodynamic force required to prevent adhesion on a surface is lower than that required for detachment. This indicates that the bond between a substratum surface and a bacterium becomes stronger after initial adhesion. Also it is more difficult to detach bacteria from dimethyldichlorosilane-coated glass (hydrophobic) than from hydrophilic glass surface [56]. We also observe that attachment is less on hydrophilic polymer such as PU, when compared to attachment on PC which is the most hydrophobic. Force required for adhesion of *E. coli* on a surface depends on the surface hydrophobicity. For example force required for *E. coli* to attach on protein coating, a hydrophobic surface, quartz, and silicon is 0.2, 3.1–4.6, 1.3–2.4 and 7400 to 22800 pN respectively.

These nanocomposites appear to be suitable for the design of implant material. Ag appears to be a better antibiofilm agent than Au. The nanoparticle in the NC prepared by swelling method will be well entrapped and bound to the polymer and hence may exhibit its antibacterial properties for long duration unlike the one prepared by coating or surface modification methods. Experiments over extended periods need to the performed to ascertain whether such NCs can be used for longer periods of time. These polymers are commonly used in medical implants and one needs to study whether the incorporation of nanoparticles has altered their biocompatibility.

## Supporting Information

File S1 **Includes Figure S1. Figure S2, and Figure S3. Figure S1. UV-Visible spectrum of Silver nanoparticle. Figure S2. UV-Visible spectrum of gold nanoparticle. Figure S3. FTIR spectra gold nanocomposites prepared by casting method. (A) PU, (B) PCLm, (C) PC and (D) PMMA.**
(DOC)
