# Supplementary material for: Antibiofilm Properties of Silver and Gold Incorporated PU, PCLm, PC and PMMA Nanocomposites under Two Shear Conditions
Source: PLoS One. 2013 May 13;8(5):e63311. doi: 10.1371/journal.pone.0063311 (PMC3652832; doi:10.1371/journal.pone.0063311)

**Supplementary Information**

**Figure S1:** UV-Visible spectrum of Silver nanoparticle

**Figure S2:** UV-Visible spectrum of gold nanoparticle

**Figure S3:** FTIR spectra gold nanocomposites prepared by casting method. (A) PU, (B) PCLm, (C) PC and (D) PMMA.


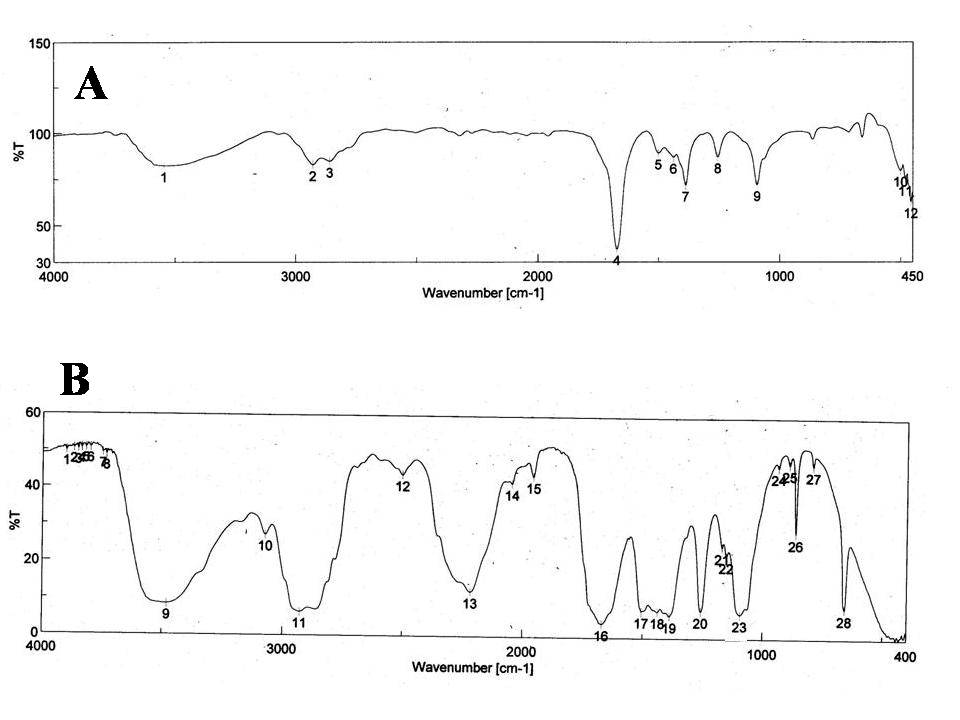


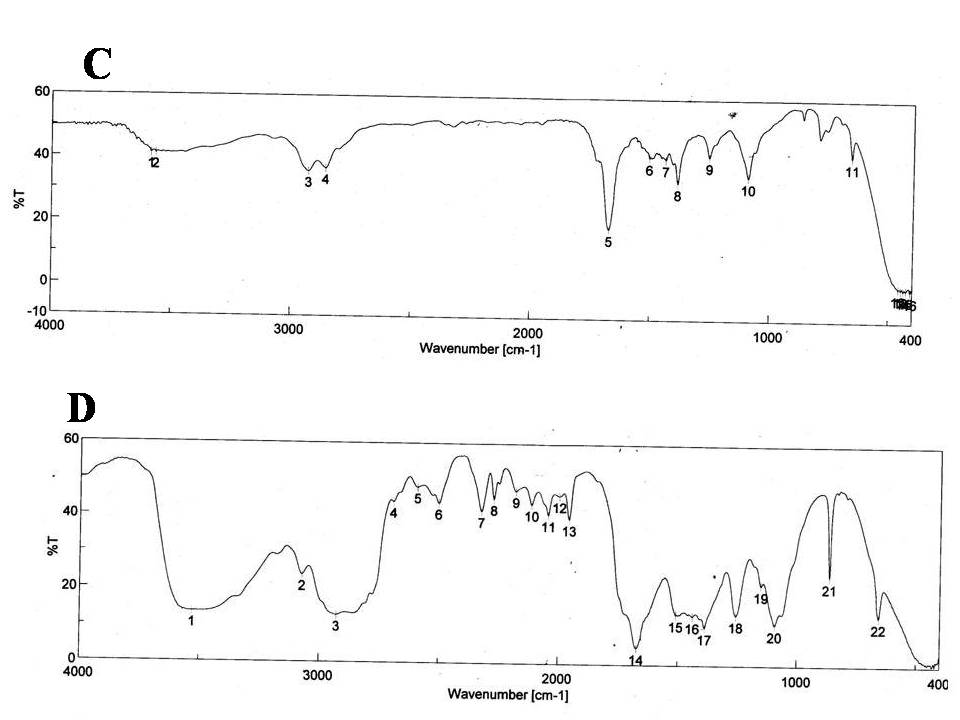

Supplement: File S1 — Includes Figure S1. Figure S2, and Figure S3. Figure S1. UV-Visible spectrum of Silver nanoparticle. Figure S2. UV-Visible spectrum of gold nanoparticle. Figure S3. FTIR spectra gold nanocomposites prepared by casting method. (A) PU, (B) PCLm, (C) PC and (D) PMMA. (DOC) [file pone.0063311.s001.doc]
